# Long-term survival in extensive-stage small-cell lung cancer treated with different immune checkpoint inhibitors in multiple-line therapies: A case report and literature review

**DOI:** 10.3389/fimmu.2022.1059331

**Published:** 2022-11-30

**Authors:** Xu Zhang, Jiabin Zheng, Yun Niu, Chongxiang Xue, Yixuan Yu, Kexin Tan, Huijuan Cui

**Affiliations:** ^1^ Graduate School, Beijing University of Chinese Medicine, Beijing, China; ^2^ Department of Integrative Oncology, China-Japan Friendship Hospital, Beijing, China; ^3^ Department of Pathology, China-Japan Friendship Hospital, Beijing, China

**Keywords:** ES-SCLC, long-term survival, ICIs, re-challenge, combined regimens

## Abstract

**Background:**

Extensive-stage small-cell lung cancer (ES-SCLC) is highly malignant, is highly prone to recurrence, and has a short survival period. It is very difficult to achieve long-term survival in ES-SCLC, which has not been significantly improved in the last 20 years. For a long time, platinum-based chemotherapy has occupied the core position in the treatment of small-cell lung cancer (SCLC), but there are few options for treatment drugs or regimens, and if disease progression occurs, the options for follow-up regimens are obviously limited. The advent of immunotherapy has changed this situation to some extent, and immunotherapy has shown some effects in improving efficiency and prolonging survival, whether in first- or third-line therapy, but it is still unsatisfactory.

**Case presentation:**

A 57-year-old patient with ES-SCLC experienced disease progression after four lines of treatment including synchronous radiotherapy, chemotherapy, and antiangiogenesis. However, the patient still benefited when switching to the programmed cell death receptor-1 (PD-1) inhibitor toripalimab in combination with chemotherapy in the fifth line. Even after the development of immune resistance, the patient still benefited after switching to tislelizumab in combination with different chemotherapy regimens or alone in the sixth and seventh lines. Following the progression of tislelizumab in combination with chemotherapy, the patient again profited after switching to durvalumab in combination with anlotinib and again achieved a progressive-free survival (PFS) of 11 months. Overall, the patient achieved a total of 45 months of PFS and 50 months of overall survival (OS), with a shocking and exciting 30 months of PFS achieved in the immune combination phase alone.

**Conclusion:**

We report a patient with ES-SCLC who achieved long-term survival after at least eight lines of therapy including chemotherapy, antiangiogenesis, and different immune checkpoint inhibitors (ICIs). This suggests that long-term survival in SCLC is possible with aggressive, combined, and standardized treatment. Otherwise, immunotherapy postline enablement can still benefit patients, rechallenge after immune resistance is also possible in SCLC, and combination with chemotherapy or antiangiogenic therapy can improve the efficacy and prolong the survival. This will provide new ideas and options for the selection of treatment options for SCLC.

## Introduction

Lung cancer, accounting for about 18% of cancer-related deaths, remains the leading cause of cancer-related deaths worldwide (1), of which small-cell lung cancer (SCLC) accounts for only 15% of lung cancer (2). However, SCLC is the most malignant type of lung cancer and has the worst prognosis, with an average overall survival (OS) of only 2–4 months in the natural course (3). On the one hand, due to its rapid proliferation rate and easy early metastasis, two-thirds of patients are in the extensive stage at the initial diagnosis, leading to its high mortality rate (4).

For extensive-stage small-cell lung cancer (ES-SCLC), comprehensive medical treatment is top ranked. SCLC is extremely sensitive to chemotherapy, and chemotherapy has excellent efficacy (2, 5). However, SCLC is very easy to relapse, and the recurrence rate within 1 year after first-line treatment is more than 80% (6). After relapse, the therapeutic effect is limited, and despite years of exploration, no more effective therapeutic drugs have emerged. Even with the advent of immunotherapy in recent years (7–10), there has been a modest improvement in the efficiency and survival of SCLC treatment with an objective response rate (ORR) of only 10% for single-agent immunotherapy in third-line treatment. Even in combination with ipilimumab, the ORR is only 33% and the maximum median progressive-free survival (PFS) is only 2.6 months (9). While in first-line treatment, the median OS was prolonged by less than 3 months compared to chemotherapy, despite an ORR of 68% for immune combination chemotherapy (11). If the disease progresses again after immunotherapy, the follow-up treatment options will also be significantly limited.

Here, we reported a case of a patient with ES-SCLC who received three different immune checkpoint inhibitors (ICIs) in combination with chemotherapy or antiangiogenic targeted therapy after progressing on fourth-line chemotherapy and achieved a total of 45 months of PFS and 50 months of high-quality OS. Such treatment results were very different from clinical reports and brought us a very great surprise.

## Case presentation

**Figure 1:**
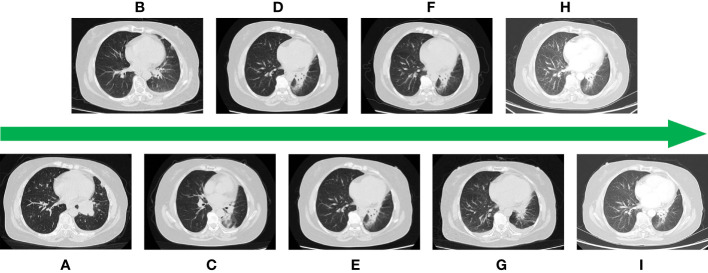
**(A)** CT image at the time of initial diagnosis. **(B)** Image after first-line treatment. **(C)** Image after second-line treatment. **(D)** Image after third-line treatment. **(E)** Image after fourth-line treatment. **(F)** Image after fifth-line treatment. **(G)** Image after sixth-line treatment. **(H)** Image after seventh-line treatment. **(I)** Image after eighth-line treatment.

**Figure 2:**
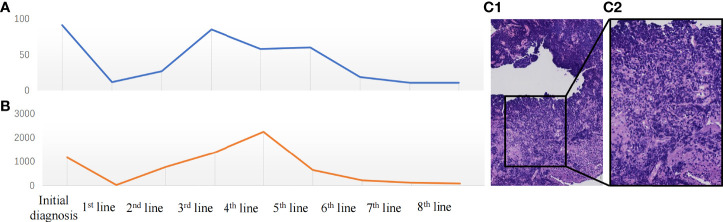
**(A)** Change in neuron-specific enolase (NSE) (ng/ml) during the treatment period. **(B)** Change in pro-gastrin-releasing peptide (Pro-GRP) (pg/ml) during the treatment. **(C)** The microscopic image of the tumor: ×200 (C1), ×400 (C2).

In January 2018, a 57-year-old Chinese woman was admitted to our hospital for hemoptysis. The patient was in good health and had no history of smoking, a family history of hereditary disease, or tumor. However, the current chest computed tomography (CT) and positron emission tomography (PET)-CT suspected left-sided advanced central-type lung cancer with multiple lymph node metastases in the mediastinum and hilar and pleural effusion (**Figure 1**). Blood tests show a significant elevation of tumor markers including neuron-specific enolase (NSE) (**Figure 2A**) and pro-gastrin-releasing peptide (Pro-GRP) (**Figure 2B**) than the normal. Fortunately, the brain magnetic resonance imaging (MRI) showed no brain metastases. Then, she accepted the fiberoptic bronchoscopy and biopsy at the same time (**Figure 2C**). Eventually, the diagnosis of ES-SCLC, cT2bN3M1a, stage IVa was given. Immunohistochemical analyses suggested “CD56 (+), CgA (±), Syn (+), CK (AE1/AE3) (perinuclear punctate +), CK5/6 (–), CK7 (-), NapsinA (-), TTF-1 (+), Ki-67 (80%+), and programmed cell death receptor ligand-1 (PD-L1) <5%.” The genetic testing demonstrated the tumor mutational burden (TMB) of 1.82 and microsatellite stabilization (MSS).

**Figure 3:**
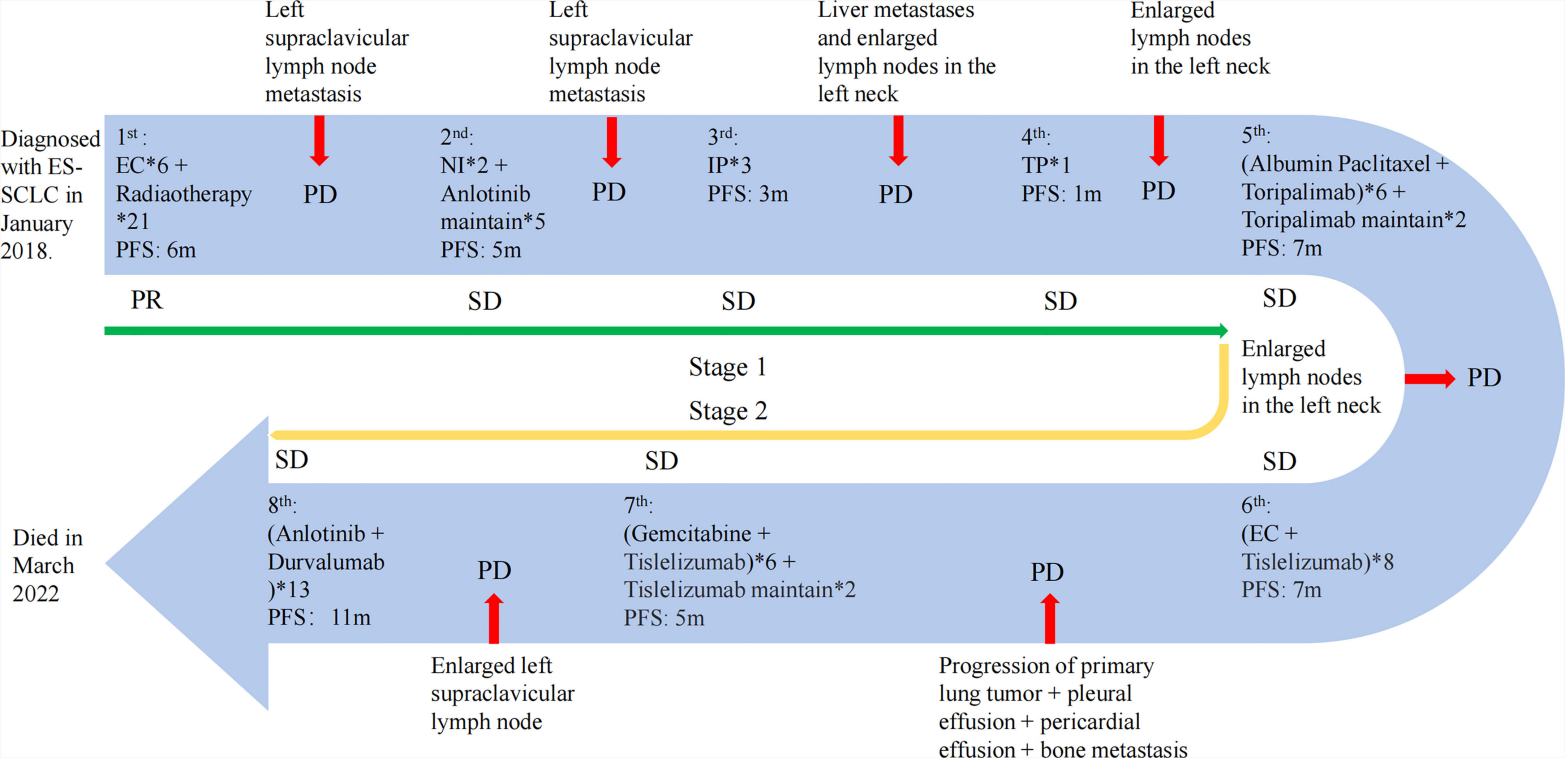
The treatment progress of the patient. The first stage includes the first to fourth lines of treatment; the fifth to eighth lines of treatment are the second stage. PR, partial response; PD, progressive disease; SD, stable disease. The efficacy evaluation was judged according to the Response Evaluation Criteria in Solid Tumours (RECIST) (Version 1.1).

She was administered four-line systematic chemotherapies including etoposide and carboplatin (EC) (**Figure 3**), vinorelbine and ifosfamide (NI) followed by anlotinib (**Figure 3**), irinotecan and pobaplatin (IP) (**Figure 3**), and albumin paclitaxel combined with cisplatin (TC) (**Figure 3**). Otherwise, she also got radiotherapy during the initial treatment period. Her disease ultimately progressed while on these systematic therapies, although some lesions shrank or were even partially relieved within a short period.

Notably, the levels of NSE (**Figure 2A**) and Pro-GRP (**Figure 2B**) were rising, and the ultrasonography and CT scan showed that the left clavicle lymph nodes were enlarged. Then, she received the fifth-line treatment with albumin paclitaxel plus programmed cell death receptor-1 (PD-1) inhibitor toripalimab (**Figure 3**). In January 2020, she received radiofrequency ablation treatment for her metastatic supraclavicular fossa lymph node lesion. However, albumin paclitaxel had to be suspended for high brain natriuretic petide (BNP) level and cardiac toxicity. Afterward, she continued receiving toripalimab alone as the maintenance treatment (**Figure 3**).

In March 2020, the touchable swollen lymph nodes on the left side of the neck revealed that the disease may have progressed. Immediately afterward, she completed ultrasound examination of neck lymph nodes and blood tumor markers, and the disease was judged to have progressed again. Thus, she started the sixth-line treatment with etoposide plus carboplatin and tislelizumab (**Figure 3**). The following CT scan revealed that the lesion has slightly progressed and she newly acquired pleural and pericardial effusion. Additionally, the bone scan of the body showed multiple bone metastases.

From that time, she received the seventh-line gemcitabine plus tislelizumab (**Figure 3**). Unfortunately, owing to the coronary heart disease and percutaneous coronary intervention, she delayed receiving the tislelizumab monotherapy then (**Figure 3**).

With a PFS of 5 months, her left supraclavicular lymph node was evaluated to be larger than before revealed by the CT scan. As a result, a change to PD-L1 agents was determined considering the demand of the patient and her family. Fortunately, she benefited from the eighth-line anlotinib plus durvalumab therapy for up to 11 months (**Figure 3**). In March 2022, she died of acute heart attack.

## Discussion

Although this patient has passed away, it is still exciting to review the complete treatment of this case (**Figure 3**). To summarize, this patient has several very distinctive features. First, this patient experienced eight lines of treatment and achieved a very long survival without a significant impact on quality of life despite being initially diagnosed with ES-SCLC. Furthermore, this patient could benefit from postline combination immunotherapy despite having received four lines of chemotherapy up front. Even after the development of immune resistance, the benefit continued after switching to a different ICI, with the patient gaining a total of 30 months of PFS during the immunotherapy phase.

In terms of ES-SCLC, the prognosis is poor and survival is short, with a median OS of only 6–10 months even after aggressive and standardized treatment (12), and a 5-year survival rate of less than 2% (13). However, this patient achieved a high-quality long-term survival of almost 50 months after multiple lines of therapy, which is exceedingly rare. A review of the literature revealed that patients with ES-SCLC with better physical status (14, 15), sensitivity to platinum-based drugs and the absence of liver or brain metastases (16), and adherence to active close follow-up may have a better prognosis with the possibility of achieving long-term survival. In these respects, the present patient is in accordance, and this may be one of the reasons for her long-term survival. However, data on long-term survival in ES-SCLC are relatively scarce (17) and are mostly single case reports (18–24), and the specific mechanisms still need to be further explored.

The infiltration of immune cells in the tumor microenvironment (25, 26) and the expression of PD-L1 (27) may be closely related to the efficacy of immunotherapy. Meanwhile, a cohort study found that the infiltration of immune cells in the tumor microenvironment may be crucial for the long-term survival of SCLC, especially the apparent increase in the number of CD3^+^ T cells, CD4^+^ T cells, CD14^+^ T cells, and tumor-infiltrating monocytes and the decrease in suppressor immune cells (28). However, this patient has not been tested for lymphocytes in the immune microenvironment, so there is no direct evidence to support this. However, by reviewing the entire treatment course and outcome of this case, we may be able to refute this hypothesis as well.

Firstly, this patient had excellent efficacy during the immunotherapy phase, although the ICI was only started from the fifth line of treatment. After progressing on fourth-line therapy, the patient first switched to toripalimab in combination with albumin paclitaxel and obtained a PFS of 7 months, with a significantly longer effective time compared to the literature, which may be related to the induction of apoptosis of tumor cells by prior chemotherapeutic agents, increased release of tumor antigenic substances, removal of immunosuppression, modulation of immune response, and remodeling of the immune microenvironment, resulting in immune potentiation (29–34).

After progressing through the fifth line of treatment, the patient switched to the PD-1 inhibitor tislelizumab in combination with different chemotherapy regimens as the sixth and seventh lines of treatment and achieved another 12 months of PFS, again surprising us with such efficacy. A previous study found that after progression on one PD-1 inhibitor in non-small-cell lung cancer (NSCLC), patients can still benefit from swapping to another PD-1 inhibitor (35). As far as we know, however, this is the first report in SCLC. In the case of this patient, we can attribute to the difference in the mechanism of the different drugs. Although both are humanized immunoglobulin G4 (IgG4) monoclonal antibodies that block the binding of PD-1 to PD-L1 or programmed cell death receptor ligand-2 (PD-L2), there are still subtle differences in the mechanism between toripalimab and tislelizumab. Toripalimab binds to PD-1 on the surface of T cells *via* the FG loop (36) while tislelizumab through the CC’ loop (37), and the dissociation rate from PD-1 is slower (37), resulting in a higher targeting affinity. In terms of pharmacokinetics, the half-life of tislelizumab is longer than that of toripalimab (38, 39).

In addition, the role of combination chemotherapy cannot be ignored (40–42), as in this case after progression of the tislelizumab combined with EC regimen, seventh-line therapy in combination with gemcitabine resulted in a renewed benefit for the patient and a significantly prolonged PFS compared to chemotherapy alone (43) or immunotherapy. It is also suggested that different chemotherapeutic agents can modify the tumor microenvironment through different mechanisms and add to the effectiveness of immunotherapy (44, 45). Unfortunately, despite the seven lines of treatment, the disease still progressed.

It is reported that when blocking the PD-1/PD-L1 signaling pathway, PD-L1 inhibitors are more effective than PD-1 inhibitors (46). In NSCLC, switching to PD-L1 inhibitor therapy after progression on PD-1 inhibitor therapy still results in disease control rate (DCR) of more than 30% and PFS can be extended by about 3 or 4 months (47–49); patient benefit has also been reported in triple-negative breast cancer (50). This suggests to us that the choice of PD-L1 inhibitor after PD-1 inhibitor progression might be a valid option. However, there are no similar reports in SCLC. In this case, after switching to PD-L1 inhibitor therapy in combination with anlotinib as eighth-line therapy, the patient again benefited with a significant prolongation of PFS for a total of approximately 11 months, which provides clinical evidence for the replacement in SCLC with PD-L1 inhibitors after progression with PD-1 inhibitors. As for the specific grounds for the benefit, we speculate that it is most likely due to the discrepancy in the modes of action between PD-1 and PD-L1 (51, 52).

However, what we still cannot ignore is the combined effect of the antiangiogenic drug anlotinib. ICIs combined with antiangiogenic therapy have been shown to improve the efficacy and prolong PFS and OS (53–56), which may be related to the fact that antiangiogenic drugs can inhibit tumor angiogenesis, reduce the blood supply to the tumor, and alter the tumor microenvironment, thus inhibiting tumor growth. Anlotinib, an essential antiangiogenic agent, has clinically proven efficacy and safety in SCLC (57, 58). The patient achieved a long PFS in this line treatment, significantly longer than reported in the literature (9, 59), which must be attributed to the synergistic effect of the immune combination with antiangiogenesis.

Reviewing the entire course of this patient’s treatment, we can observe that the patient obtained a very long and high-quality survival. Especially in the immunotherapy phase, the sequential application of different ICIs and the combination of different regimens brought the possibility of long-term survival for the patient.

In the era of chemotherapy for SCLC, switching to another chemotherapy regimen after progression has become a routine option. While entering the era of immunotherapy, whether it is possible to sequentially apply different ICIs after the progress of one or more ICI therapies has not been reported. However, this case provides objective evidence for the efficacy and safety of immune rechallenge in SCLC.

## Conclusion

First, after aggressive and standardized treatment and close follow-up, long-term benefit is still possible, even in SCLC. In addition, immunotherapy remains effective in the later line of treatment. Even if immune resistant, the patient could still benefit again after changing ICIs; and the efficacy could be further improved by combining different treatment regimens. This provides new ideas and options for the treatment process of clinically ES-SCLC.

## Data availability statement

The original contributions presented in the study are included in the article/supplementary material. Further inquiries can be directed to the corresponding author.

## Ethics statement

Written informed consent was obtained from the individual(s) for the publication of any potentially identifiable images or data included in this article.

## Author contributions

XZ and JZ: Collect clinical data, accomplish and revise the paper. YN: Pathology consultation and diagnosis. CX: Revise the paper and organize the paper. YY: Check clinical data and revise the paper. KT: Organize paper. HC: Provide cases, describe the novelty and particularity of the cases, review and revise the content of the article. All authors contributed to the article and approved the submitted version.

## Funding

This study was supported by the National High Level Hospital Clinical Research Funding (2022-NHLHCRF-LX-02-0111) and the Capital Health Development Research Project (shoufa 2022-2-4065).

## Conflict of interest

The authors declare that the research was conducted in the absence of any commercial or financial relationships that could be construed as a potential conflict of interest.

## Publisher’s note

All claims expressed in this article are solely those of the authors and do not necessarily represent those of their affiliated organizations, or those of the publisher, the editors and the reviewers. Any product that may be evaluated in this article, or claim that may be made by its manufacturer, is not guaranteed or endorsed by the publisher.
